# Diagnostic performance of discriminant formulas and machine learning models for detecting β-thalassemia trait in Bangladesh

**DOI:** 10.1371/journal.pone.0350387

**Published:** 2026-06-16

**Authors:** Rumana Mahtarin, Kasrina Azad, Rakib Bin Mahbub Talukder, Rynak Rahmat, Suzana Chowdhury Nitu, Arif Mahmud Howlader, Mohabbat Hossain, Mst. Sharmin Aktar Mukta, Mohammad Tanbir Habib, Abu Bakar Siddik, Nishat Sultana, Zannat Kawser, Umme Kulsum, Faisal Zainal Abedin, Nusrat Sultana, Md. Ahashan Habib, A. K. M. Ekramul Hossain, Farjana Akther Noor, Ahmad Zubair Mahdi, Muhammad Asaduzzaman, Emran Kabir Chowdhury, Md Rofiqur Rahman, Firdausi Qadri, Mst. Noorjahan Begum, A. H. M. Nurun Nabi

**Affiliations:** 1 Institute for Developing Science and Health Initiatives (ideSHi), Dhaka, Bangladesh; 2 Department of Biochemistry and Molecular Biology, University of Dhaka, Dhaka, Bangladesh; 3 Department of Electronics and Communication Engineering (ECE), Khulna University of Engineering and Technology (KUET), Khulna, Bangladesh; 4 Department of Genetic Engineering and Biotechnology, University of Chittagong, Chittagong, Bangladesh; 5 Department of Microbiology, Popular Medical College, Dhaka, Bangladesh; 6 Department of Virology, Dhaka Medical College, Dhaka, Bangladesh; 7 Directorate General of Health Services, Dhaka, Bangladesh; 8 Department of Project Development, Bangladesh Thalassemia Samity and Hospital, Dhaka, Bangladesh; 9 Molecular Division, OMC Healthcare (Pvt.) Ltd, Rupnagar, Dhaka, Bangladesh; 10 Department of Community Medicine and Public Health, Tairunnessa Memorial Medical College and Hospital, Gazipur, Bangladesh; 11 Upazila Health Complex, Matlab (North), Chandpur, Bangladesh; 12 Mucosal Immunology and Vaccinology, Infectious Diseases Division, International Centre for Diarrhoeal Disease Research, Mohakhali, Dhaka, Bangladesh; 13 Virology Laboratory, Infectious Diseases Division, International Centre for Diarrhoeal Disease Research, Mohakhali, Dhaka, Bangladesh; National Research Centre, EGYPT

## Abstract

**Background:**

β-thalassemia poses a considerable public health burden in Bangladesh, where a high carrier frequency underlies widespread disease risk. It is necessary to distinguish β-thalassemia trait (βTT) and iron deficiency anemia (IDA) to ensure genetic counseling and enable effective prevention strategies. Despite the availability of various discriminant formulas and machine learning algorithms (MLAs), their comparative diagnostic performance within the Bangladeshi population has not been comprehensively investigated. This study aimed to assess different discriminant formulas and ML models as well as to propose novel combinations of formulas for population-specific screening of βTT.

**Methods:**

In this cross-sectional study, we compared 47 discriminant formulas and 12 machine learning models to distinguish β-thalassemia trait from iron-deficiency anemia in 467 individuals (143 βTT, 324 anemia) drawn from a 2,514-participant cohort. DF-6 and DF-27 were two new formulas constructed by integrating high-performing formulas. Multi-criteria decision-making (MCDM) techniques, TOPSIS (Technique for Order Preference by Similarity to Ideal Solution) and SECA (Simultaneous Evaluation of Criteria and Alternatives), provided the final ranking for performance. Cluster analysis was performed to identify groups with similar diagnostic performance.

**Results:**

Population-specific optimal cut-off values were determined for the discriminant formulas. The newly proposed formulas, DF-6 and DF-27, ranked among the top ten performers alongside RBC, Janel (11T), Ravanbakhsh-F1, Srivastav, Alparslan, Hisham, Index 26, and Kerman I. DF-6 (AUC: 0.9707) achieved the best overall performance across the diagnostic metrics. DF-6 achieved the best overall performance (AUC: 0.98, 95% CI: 0.97–0.99, p < 0.0001). Assessment of ML models revealed that XGBoost (XGB) (AUC: 0.98, 95% CI: 0.97–0.99, p < 0.0001) and Support Vector Machine (SVM) (AUC: 0.97, 95% CI: 0.95–0.99, p < 0.0001) provided the highest diagnostic accuracy. The reliability of ensemble ML models was confirmed by MCDM and cluster analyses.

**Conclusions:**

The combination of novel discriminant formula DF-6 and integration of XGB and SVM ML models can substantially strengthen nationwide screening programs to reduce the burden of thalassemia in Bangladesh.

## 1. Introduction

Thalassemia refers to a group of inherited blood disorders characterized by defective synthesis of globin chains. Among its types, β-thalassemia results from mutations that reduce or abolish the production of β-globin chains [1]. People with β-thalassemia major (β0/β0, β0/β + , and sometimes β + /β+) generally receive medical attention within the first two years of life. They necessitate regular red blood cell transfusions to survive [2]. The disorder is particularly concerning in countries within the global thalassemia belt [3]. Bangladesh lies within this belt, where β-thalassemia and hemoglobin E (HbE) are widespread [4–7]. The frequency of thalassemia carriers ranges from 10.9% to 13.3% [8]. The challenge is compounded by iron deficiency anemia (IDA), which often mimics β-thalassemia trait (βTT) in hematological profiles [9–11]. In such settings, early and accurate detection of carriers is essential to enable appropriate genetic counseling and break the chain of transmission. Gold-standard diagnostic methods such as hemoglobin electrophoresis, molecular analysis, iron profile provide definite results [5,7,9]. However, the cost and logistical demands make their uses unsuitable for large-scale screening programs. To overcome these barriers, various diagnostic formulas have been proposed worldwide, which are derived from complete blood count (CBC) indices [12–14]. These formulas offer a rapid and inexpensive preliminary screening approach. However, their performance varies across populations due to differences in demographics, genetic backgrounds, and study methodologies [9]. Therefore, optimization of population-specific cut-off values for those formulas is mandatory. In parallel, machine learning algorithms (MLAs) have emerged as powerful tools for analysis of biomedical data. Classical classifiers (Logistic Regression, Decision Tree, Random Forest, Support Vector Machine, Multilayer Perceptron, Linear Discriminant Analysis, K-Nearest Neighbors, and Gaussian Naive Bayes), as well as ensemble methods (Gradient Boosting, AdaBoost, XGBoost, and CatBoost), have improved diagnostic accuracy in thalassemia by identifying complex and nonlinear patterns within biomedical datasets [12,15–21]. Moreover, multi-criteria decision-making (MCDM) techniques show high capacity to improve risk assessment of thalassemia. While traditional formula ranking lack multi-criteria evaluation, it fails to capture the highest performance [12,13,22].

However, there is limited literature of comparative analyses combining conventional indices and various machine learning algorithms in Bangladeshi cohort. In addition, the use of structured multi-criteria assessment systems has been rarely used to rank diagnostic methods in a systematic manner using several performance indicators at the same time. This methodological gap prevents the evidence-based choice of the best screening methods to be applied to the large-scale population [23,24]. Therefore, this study aimed to: (1) optimize cut-off values of existing discriminant formulas for detecting βTT in the Bangladeshi population; (2) derive and evaluate two new composite indices (DF-6, DF-27); (3) compare their performance with multiple MLAs based on routine CBC indices; (4) use multi-criteria decision-making methods such as TOPSIS and SECA to offer a systematic ranking of diagnostic tools as a screening method; and (5) group data points based on similarity using agglomerative hierarchical clustering.

Consequently, the study justifies the implementation of evidence-based and resource-efficient screening approaches in Bangladesh to reduce burden of thalassemia and improve patient care outcomes.

## 2. Methods

### 2.1. Enrollment of study participants and ethical clearance

In this cross-sectional study, blood samples were collected from 2,514 individuals through thalassemia carrier screening programs conducted at various sites in Bangladesh, including universities, medical colleges, and specialized hospitals, such as Bangladesh Thalassemia Samity Hospital, Kurmitola General Hospital, from 20 April 2022 to 31 March 2023. The samples were analyzed at the laboratory of Institute for Developing Science and Health Initiatives (ideSHi). This study was ethically approved under a research protocol (# PNR-22003) by the Institutional Review Board of the Institute for Developing Science and Health Initiatives (ideSHi). Participants provided informed assent or consent, and written consent was obtained from the participants, their parents, or legal guardians.

### 2.2. Analysis of complete blood count of the study participants

Two milliliters of venous blood were collected from each participant in an EDTA-containing vacutainer, and the collected blood samples were transported to the laboratory, maintained at 4–8°C, and preserved at 4°C until analysis. A complete blood count (CBC) was performed for each sample by an XS-800i Hematology Analyzer (Sysmex, Japan) following the manufacturer’s instructions. Major hematological parameters, such as hemoglobin (HGB), hematocrit (HCT), red blood cell (RBC) count, and red cell indices, including the mean corpuscular volume (MCV), mean corpuscular hemoglobin (MCH), mean corpuscular hemoglobin concentration (MCHC), and red cell distribution width (RDW), were considered for analysis.

### 2.3. Hemoglobin electrophoresis

Hemoglobin electrophoresis was performed on an automated capillary electrophoresis system (Sebia, France) using a capillary hemoglobin (E) kit to measure HbA, HbA2, HbF, and other abnormal Hb variants following the manufacturer’s instructions.

### 2.4. Screening of the population for diagnostic performance analysis

**Fig 1:**
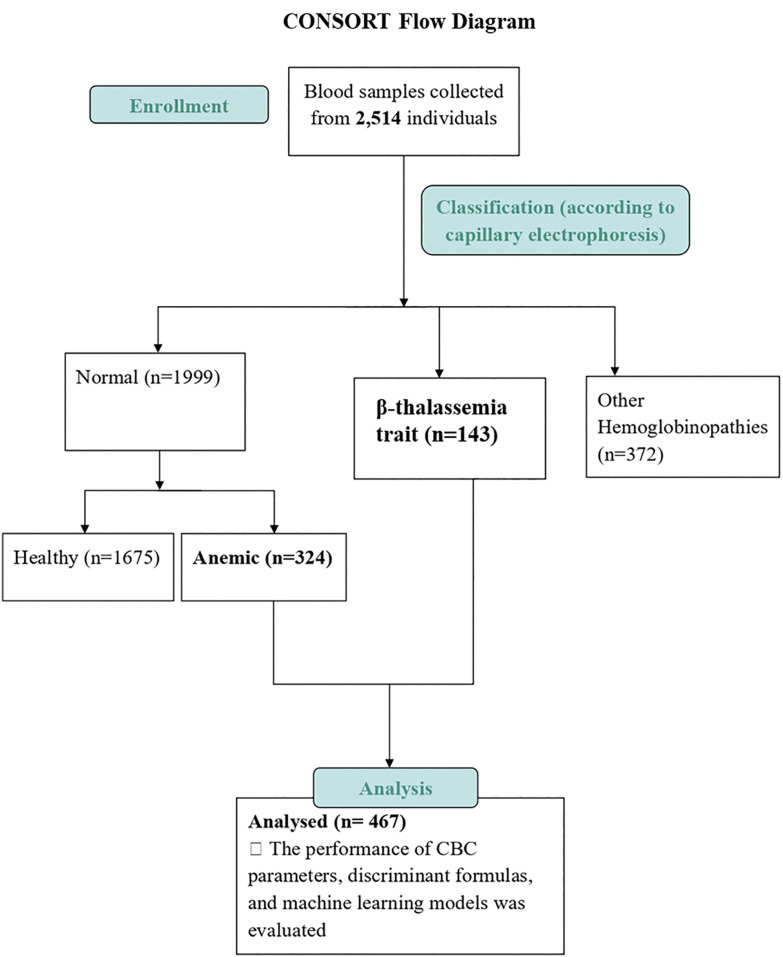
Study design for diagnostic performance analysis.

Among the 2,514 samples, hemoglobin electrophoresis determined 1999 to be normal,143 had beta thalassemia trait (βTT), 5 had delta-beta thalassemia trait (δ-βTT), 324 had hemoglobin E trait (HbET), 7 had HbE disease, 11 had hereditary persistence of fetal hemoglobin (HPFH), 8 had hemoglobin D (HbD), 4 had hemoglobin S (sickle cell trait), 1 had hemoglobin H (HbH), 1 had beta thalassemia major (βTM), 5 had HbE-beta thalassemia (HbE-βT), 2 had compound heterozygote of HbE-β and HbD (HbE-βT-HbD) and 4 had rare hemoglobin variants. Out of the 1999 samples, 324 were classified as anemic. The anemic group had hemoglobin (HGB) levels <12 and <13 g/dL for women and men, respectively. MCV and MCH were <80 fL and <27 pg for both genders, respectively. The βTT group had an MCV value <80 fL and an MCH value <27 pg. HbA2 > 3.5% was considered as βTT. The selection and classification were conducted in accordance with the WHO diagnostic guidelines [25]. Diagnostic performance analyses of formulas and ML models focused on the 467 individuals (143 βTT, 324 anemia). Fig 1 shows overview for sampling strategy and final selection of analytic datasets.

### 2.5. Optimization of population specific cut-off values for discriminant formulas

For each discriminant formula, the optimal population-specific cut-off value was determined using receiver operating characteristic (ROC) curve analysis in the analytic dataset. The optimal cut-off value was selected by maximizing Youden’s Index (J) which is given in (1).


$$\begin{array}{c} J=Sensitivity+Specificity-1 \end{array}$$ (1)

where sensitivity represents the true positive rate and specificity represents the true negative rate. The cut-off value corresponding to the maximum J was considered optimal [9].

### 2.6. Derivation and validation of new discriminant formulas

Along with the existing 45 CBC derived discriminant formulas, two new formulas, DF-6 and DF-27, were introduced to improve diagnostic performance, by combining the high performing established formulas (Table 1). For each constituent formula, a binary classification score was assigned shown in (2).

**Table 1:** Discriminant formulas applied for the evaluation of the diagnosis of β-thalassemia trait (βTT) in the Bangladeshi population.

Sl no.	Discriminant Formula	Calculation	Original Cut-off for βTT	Optimal Cut-off for βTT
1.	England and Fraser (E&F) [26]	MCV-RBC-(5 Hb)-3.4	<0	< 8.4
2.	RBC [9]	RBC	>5	≥4.79
3.	Mentzer [27]	MCV/RBC	<13	< 14.83
4.	Srivastava [28]	MCH/RBC	<3.8	<4.53
5.	Shine and Lal (S&L) [29]	MCV^2^ × MCH/100	<1530	< 965.41
6.	Bessman [30]	RDW	<15	≥15.2
7.	Ricerca [31]	RDW/RBC	<3.3	<3.2
8.	Green and King (G&K) [32]	MCV^2^ × RDW/100 Hb	<65	< 65.31
9.	Jayabose [33]	(MCV × RDW)/RBC	<220	< 228.85
10.	Sirdah [34]	MCV-RBC-(3 Hb)	<27	< 30.79
11.	Ehsani [35]	MCV-(10 RBC)	<15	< 22.5
12.	Sirachainan [36]	1.5 HB-0.05 MCV	>14	≥12.6
13.	Das Gupta [37]	1.89RBC-0.33RDW-3.28	>0	≥ 0.55
14.	Telmissani – MCHD [38]	MCH/MCV	<0.34	< 0.32
15.	Telmissani – MDHL [38]	(MCH × RBC)/MCV	>1.75	≥1.5
16.	Huber– Herklotz [39]	(MCH × RDW × 0.1/ RBC) +RDW	<20	<24.5
17.	Kerman I [40]	(MCV × MCH)/RBC	<300	< 326.5
18.	Kerman II [40]	(MCV × MCH × 10)/(RBC×MCHC)	<85	<97.7
19.	Keikhaei [41]	Hb × RDW × 100/ RBC^2^ × MCHC	<21	<22.9
20.	Nishad (Thal) [42]	0.615 MCV + 0.518 MCH + 0.446 RDW	<59	<60.1
21.	Wongprachum [43]	(MCV × RDW/RBC)-10 HB	<104	<131.3
22.	Sehgal [44]	MCV^2^/RBC	<972	< 976.4
23.	Sargolzaie [45]	125.643 + 44.304 × RBC-20.932 × Hb-2.501 × MCV + 20.302 × MCH-12.183 × MCHC	<0.5	<−20
24.	Pornprasert [46]	MCHC	<31	<32
25.	Plengsuree [47]	RDW/RBC	<3.3	<3.2
26.	Bordbar [48]	│80-MCV│ × │27-MCH│	>44.76	≥58.4
27.	Hisham [49]	MCH × RDW/RBC	<67	<75.5
28.	Hameed [49]	MCH × HCT × RDW/(RBC × Hb)^2^	<220	<114.5
29.	Matos and Carvalho (M&C) [50]	1.91 × RBC + 0.44 × MCHC	>23.85	≥23.3
30.	Ravanbakhsh -F1 [51]	MCV/ HCT	<2.0	<2.1
31.	Ravanbakhsh-F2 [51]	RDW-3 × RBC	<1.5	< 0.96
32.	Ravanbakhsh-F3 [51]	MCV × RDW-(100 × RBC)	<600	<565.1
33.	Ravanbakhsh-F4 [51]	MCV × Hb/ RDW × RBC	<10	<8.38
34.	Zaghloul-1 [52]	Hb + HCT + RBC	>52.5	≥47.73
35.	Zaghloul-2 [52]	Hb + HCT + RBC − RDW	>37.1	≥32.97
36.	Kandhro-1 [53]	RBC/HCT + 0.5 × RDW	<8.2	≥7.7
37.	Kandhro-2 [53]	RDW × 5/RBC	<16.8	< 15.98
38.	Merdin-1 [54]	RDW × RBC/MCV	>1.27	≥1.21
39.	Merdin-2 [54]	RDW × RBC × Hb/MCV	>14.7	≥11.3
40.	Cruise [9]	MCHC+0.603 RBC + 0.523 RDW	≥42.63	≥46.86
41.	Index 26 [9]	Combination of 26 formulas	≥ 16	≥ 14
42.	Janel (11T) [55]	Combination of 11 formulas	≥ 8	N/A
43.	Roth [14]	1.45 × (MCV − 82.8)/ 10.28 + 0.66 × (MCH − 27.0)/ 3.9 + 0.98	< 0.0	<4.44
44.	SCS_BTT_ [12]	0.2815MCV + 0.2015MCH −0.2641RBC − 0.1693RDW +0.0835Hb	<24.99	<20.27
45.	Alparslan [54]	log_10_ (MCH × MCHC × RDW/ RBC)	<3.34	<3.37
46.	DF-6	Combination of RBC, Alparslan, Hisham, Kerman II, Ravanbakhsh-F1, and Srivastava	N/A	≥4
47.	DF-27	Combination of RBC, Alparslan, Bordbar, Das Gupta, Ehsani, England and Fraser (E&F), Green and King (G&K), Hameed, Hisham, Jayabose, Keikhaei, Kerman I, Kerman II, Mentzer, Merdin-1, Merdin-2, Nishad (Thal), Ravanbakhsh-F1, Ravanbakhsh-F4, Roth, SCS_BTT_, Sehgal, Shine and Lal (S&L), Sirdah, Srivastava, TI (MDHL), and Wongprachum	N/A	≥16

Here, RBC means Red Blood Corpuscle, HGB means Hemoglobin, HCT means Hematocrit, MCV means Mean Corpuscular Volume, MCH means Mean Corpuscular Hemoglobin, MCHC means Mean Corpuscular Hemoglobin Concentration, and RDW-CV means Red Cell Distribution Width-Coefficient of Variation, DF-6 means Discriminant Formula-6, DF-27 means Discriminant Formula-27.


$$\begin{array}{c} I_{k}=\left\{ \begin{array}{c} @l\text{1}ifpredictedas\beta \text{TT}\\ \text{0}ifpredictedasIDA \end{array}\right. \end{array}$$ (2)

Where *I*_*k*_ denotes the binary output of the k-th discriminant formula.

DF-6 was derived from a combination of RBC, Alparslan, Hisham, Kerman II, Ravanbakhsh-F1, and Srivastava indices. The individual scores were then summed to obtain a final composite score. DF-6 scoring was defined in (3).


$$\begin{array}{c} DF-\text{6}=I_{RBC}+I_{Alparslan}+I_{Hisham}+I_{KermanII}+I_{\text{Ravanbakhsh}-\text{F1 }}+I_{srivastava} \end{array}$$ (3)

DF-6 ranges from 0 to 6. A DF-6 score ≥4 classified an individual as βTT.

DF-27 was derived from RBC, Alparslan, Bordbar, Das Gupta, Ehsani, England and Fraser (E&F), Green and King (G&K), Hameed, Hisham, Jayabose, Keikhaei, Kerman I, Kerman II, Mentzer, Merdin-1, Merdin-2, Nishad (Thal), Ravanbakhsh-F1, Ravanbakhsh-F4, Roth, SCS_BTT_, Sehgal, Shine and Lal (S&L), Sirdah, Srivastava, TI (MDHL), and Wongprachum. DF-27 scoring was defined as sum of these indices as shown in (4).


$$\begin{array}{c} DF-\text{2}\text{7}=\sum \overset{\text{2}\text{7}}{\underset{k=\text{1}}{\mathrm{\backslash nolimits}}}I_{k} \end{array}$$ (4)

Where *I*_*k*_ represents the binary classification outcome of each constituent formula.

DF-27 score ranges from 0 to 27. A DF-27 score ≥16 classified an individual as βTT. The optimal cut-off values were determined using ROC curve analysis by maximizing Youden’s Index. The performance of DF-6 and DF-27 was validated using 324 anemic cases and 143 βTT cases.

### 2.7. Assessment of diagnostic performance

The performance of major hematological indices from the CBC, 47 discriminant formulas, and 12 machine learning models, was assessed using the following 12 measures: sensitivity, specificity, false negative rate, false positive rate, positive predictive value, negative predictive value, Youden’s Index, accuracy, positive likelihood ratio (LR+), negative likelihood ratio (LR−), diagnostic odds ratio (DOR), and area under the curve (AUC) [9,56]. The equations were as follows:


$$\begin{array}{c} \text{ Sensitivity }(\text{Tr}\text{ue Positive Rate})=\frac{\text{True positive }}{\left(\text{True positive}+\text{False Negative}\right)} \end{array}$$ (5)

$$\begin{array}{c} \text{ Specificity }(\text{True Negative Rate})=\frac{\text{True Negative}}{\left(\text{True Negative}+\text{False Positive}\right)} \end{array}$$ (6)

$$\begin{array}{c} \text{False Negative Rate}=(\text{1}-\text{Sensitivity}) \end{array}$$ (7)

$$\begin{array}{c} \text{False Positive Rate}=(\text{1}-\text{Specificity}) \end{array}$$ (8)

$$\begin{array}{c} \text{ Positive Predictive Value }(\text{PPV})=\frac{\text{True positive }}{\left(\text{True positive}+\text{False Positive}\right)}\; \end{array}$$ (9)

$$\begin{array}{c} \text{ Negative Predictive Value }(\text{NPV})=\frac{\text{True Negative}}{\left(\text{True Negative}+\text{False Negative}\right)} \end{array}$$ (10)

$$\begin{array}{c} \text{Youden}\text{'}\text{s Index}=\text{ Sensitivity}+\text{ Specificity}-\text{1} \end{array}$$ (11)

$$\begin{array}{c} \text{Accuracy}=\frac{\text{True positive}+\text{True Negative}}{\left(\text{True positive}+\text{True Negative}+\text{False Positive}+\text{False Negative}\right)} \end{array}$$ (12)

$$\begin{array}{c} \text{ Positive Likelihood Ratio }(\text{LR}+)=\frac{\text{Sensitivity}}{\left(\text{1}-\text{Specificity}\right)} \end{array}$$ (13)

$$\begin{array}{c} \text{Negative Likelihood Ratio }(\text{LR}+)=\frac{\text{1}-\text{Sensitivity}}{\left(\text{Specificity}\right)} \end{array}$$ (14)

$$\begin{array}{c} \text{Diagnostic Odds Ratio}(\text{DOR})=\frac{\text{PositiveLikelihood Ratio}}{\text{Negative Likelihood Ratio}}\; \end{array}$$ (15)

To ensure statistical robustness and reduce optimistic bias, 95% confidence intervals (CI) were calculated for key diagnostic measures. For Sensitivity, specificity, accuracy, FPR, FNR, PPV, and NPV, confidence intervals were derived using Wilson score. For AUC, Youden Index, LR + , LR-, DOR, confidence intervals were derived using non-parametric bootstrap resampling.

#### 2.7.1. Data preprocessing.

**Fig 2:**
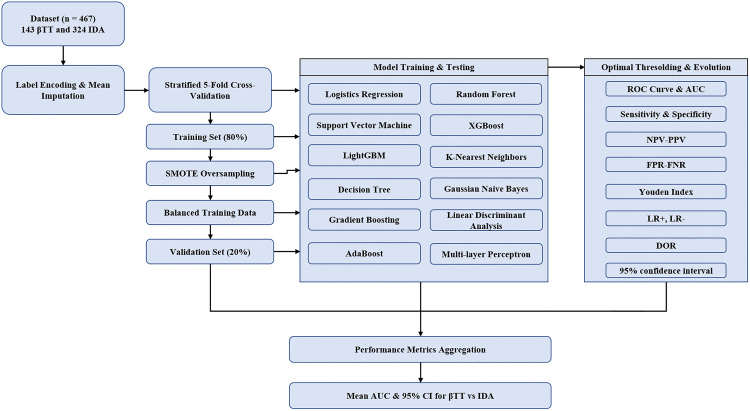
Machine learning modeling workflow.

We restricted ML analyses to the anemia vs βTT subset (n = 467) and trained binary classifiers (βTT vs anemia). In one set of models, features were CBC indices alone; in a second set, the 47 discriminant formulas were used as features. Major hematological parameters, such as HGB, HCT, RBC count, and red cell indices, including MCV, MCH, MCHC, and RDW-CV, and discriminant indices were preprocessed (Fig 2).

#### 2.7.2. Machine learning model and feature selection.

The following supervised machine learning models were selected for the assessment of diagnostic performance: Logistic Regression (LR), Random Forest (RF), Support Vector Machine (SVM), K-Nearest Neighbors (KNN), Decision Tree (DT), Gaussian Naive Bayes (GNB), Gradient Boosting Machine (GBM), XGBoost (XB), LightGBM (LGBM), Multi-layer perceptron (MLP), Linear Discriminant Analysis (LDA), and AdaBoost (ADA). CBC parameters and 47 discriminant indices were selected as feature to be analysed. Class imbalance was addressed using cost-sensitive learning (class_weight = “balanced”) where applicable.

#### 2.7.3. Cross-validation and resampling strategy.

The machine learning workflow is summarized in Fig 2. Model evaluation was performed using stratified 5-fold cross-validation to preserve class distribution across folds. In each iteration, approximately 80% of the data were used for training and 20% for validation. To mitigate class imbalance, the Synthetic Minority Over-sampling Technique (SMOTE) was applied to the training data within each fold. SMOTE resulted in an approximately balanced class distribution with a 1:1 ratio. The validation data remained untouched to prevent information leakage. This fold-wise procedure ensured that estimation of model performance was unbiased and generalizable, thereby minimized the risk of overfitting.

#### 2.7.4. Feature scaling.

Feature scaling was conducted using StandardScaler within each fold. The scaler was fitted only on the training subset and subsequently applied to the corresponding validation fold, thereby preventing data leakage.

#### 2.7.5. Model training, threshold optimization, and performance aggregation.

Within each cross-validation fold, models were trained on SMOTE-balanced training data. For probabilistic classifiers, predicted probabilities were obtained for the validation fold. Optimal classification thresholds were determined using Youden’s Index derived from ROC analysis. Binary predictions were then generated using these optimal thresholds. After completion of all folds, predictions from the validation subsets were pooled to construct a global confusion matrix for each model. Performance metrics and their corresponding confidence intervals were calculated from these pooled predictions, providing stable and unbiased overall estimates. Mean AUC values were reported with bootstrap-derived 95% confidence intervals as illustrated in Fig 2.

### 2.8. Ranking by multi-criteria decision-making (MCDM) methods

The CBC indices, discriminant formulas, and ML models were ranked by MCDM methods, TOPSIS and SECA. TOPSIS evaluated and ranked on their closeness to the ideal and anti-ideal solutions. The best solution was considered the maximum of each criterion, and the worst solution was the minimum. The TOSIS score was calculated for the ranking of the models; the higher the score, the better the results. SECA analysed multiple criteria and alternatives simultaneously by maximizing the overall performance [57–59].

### 2.9. Hierarchical clustering

The Ward linkage method was used for hierarchical clustering. This method with Euclidean distance, minimized the total variance within each cluster and created groups of formulas that were as internally homogeneous as possible in terms of their performance profiles.

### 2.10. Statistical analysis

The statistical analyses were performed using SPSS and GraphPad Prism software (version 9.0), R, and Python programming. Non-parametric and parametric tests, including the Kruskal-Wallis test, Welch’s t-test, and Mann-Whitney U test were performed. For all tests, a two-tailed P value <0.05 was considered statistically significant. Machine learning model analyses were conducted in Python using the Scikit-learn, Imbalanced-learn, XGBoost, LightGBM, and Statsmodels libraries. Bootstrap confidence interval estimation and Wilson score interval calculations were performed using SciPy and Statsmodels modules.

## 3. Results

### 3.1. Descriptive statistics

In this study, major hematological parameters, such as HGB, HCT, RBC count, and red cell indices, including MCV, MCH, MCHC, and RDW-CV, were considered for analysis of 2,514 individuals. The variation in hematological parameters among 13 groups were shown in supporting Fig 3. Performing Kruskal-Wallis test, it was found that RBC, HGB, MCV, MCH, MCHC, and RDW-CV were significantly different (p-value <0.0001) among the 13 groups. RBC and MCHC remained the highest in HbH and the lowest in βTM. HGB and MCV were the highest in the normal group, while the lowest values were observed in βTM and HbH, respectively. MCH was the highest in the HPFH group and the lowest in the HbH group. RDW-CV was the highest in HbE-βT-HbD and the lowest in normal. The comparison of the parameters in the βTT and anemic group was illustrated in Fig 4 where RBC, HGB, HCT, and RDW-CV were lower in the anemic group than in the βTT group, while MCV, MCH, and MCHC were higher in the anemic group than in the βTT group. Welch’s t-test was performed, and the p-value was statistically significant (p-value <0.0001). In hemoglobin electrophoresis, HbA and HbA2 were statistically different (p-value <0.0001) in the anemic and βTT groups. HbA2 was significantly lower in the anemic group than in the βTT group (Fig 5). Fig 6 shows the age and gender-wise distribution of individuals. Females accounted for the majority of anemia cases, as indicated by a significantly higher prevalence of anemia among females. Males showed comparatively higher proportions of βTT compared to females, as displayed by the stronger purple flows toward males.

**Fig 3:**
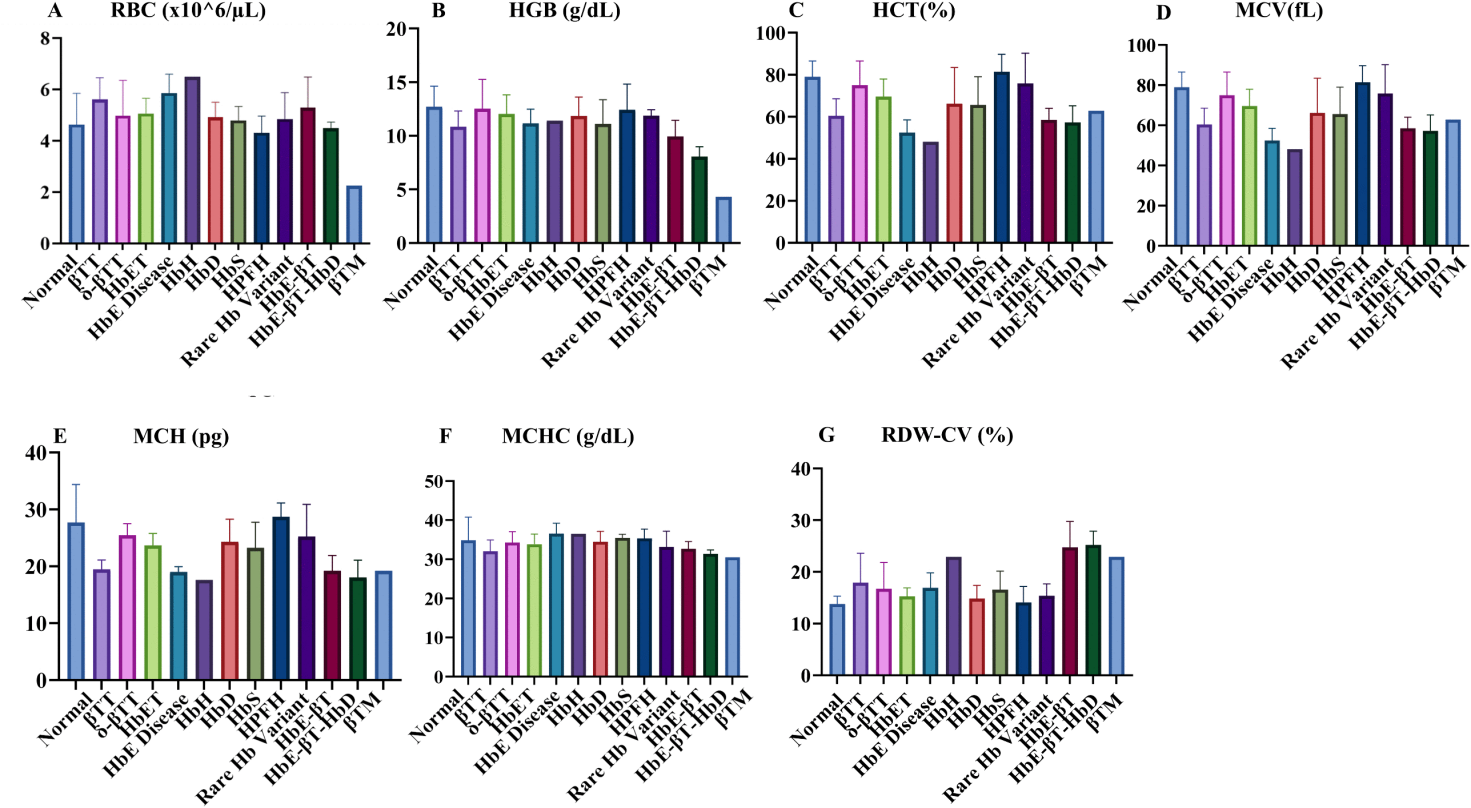
Distribution pattern of major hematological parameters in different groups.

**Fig 4:**
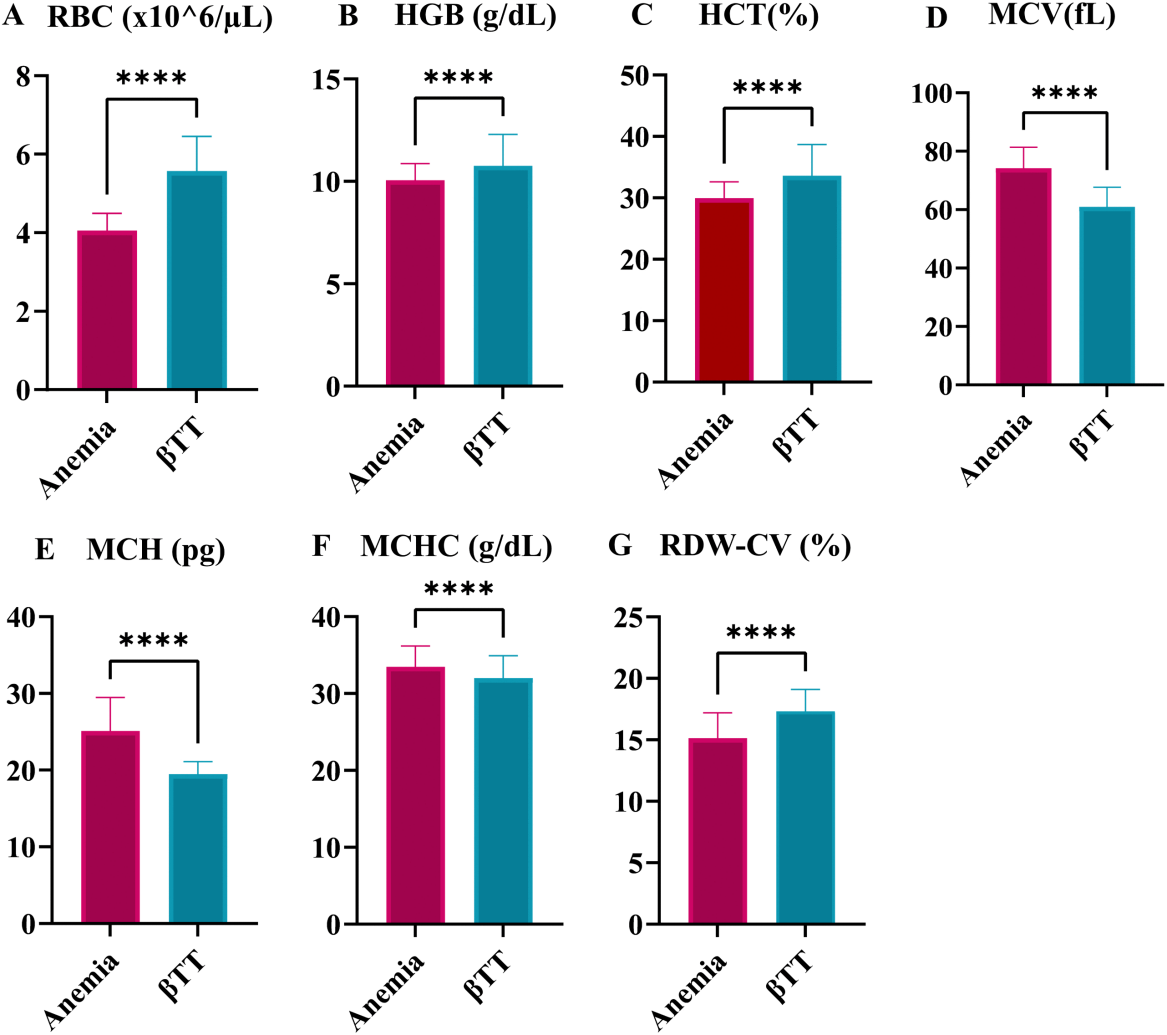
Distribution pattern of major hematological parameters in anemic and βTT groups.

**Fig 5:**
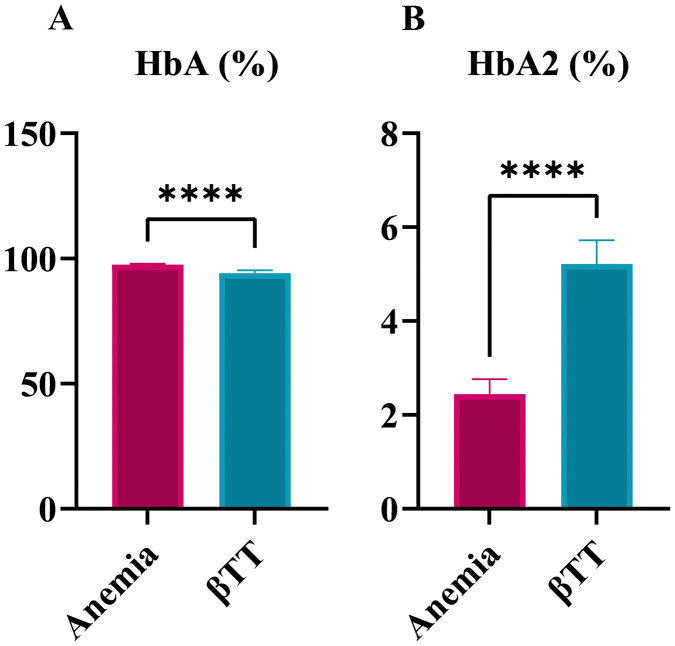
Hemoglobin electrophoresis results for anemia and βTT cases. (A) Comparison of HbA (%) in anemia and βTT cases and (B) Comparison of HbA2 (%) in anemia and βTT cases.

**Fig 6:**
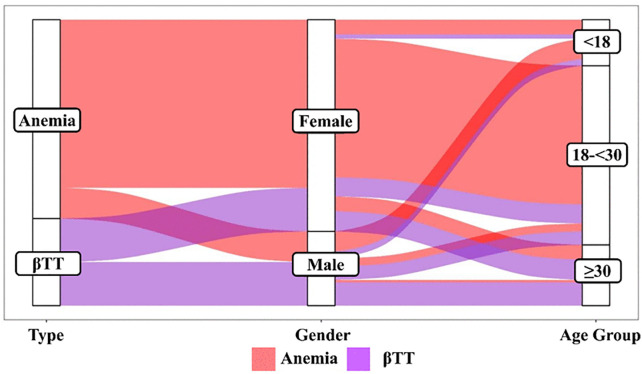
The alluvial plot represented the distribution of anemia and β-thalassemia trait (βTT) groups. The color codes indicated different groups: anemia (red), βTT (purple).

### 3.2. Assessment of diagnostic performance of CBC parameters, discriminant formulas, and machine learning models

The performance of CBC parameters, discriminant formulas, and machine learning models was evaluated for the detection of 143 βTT cases, compared with 324 anemic individuals. The cut-off values for most formulas were optimized for the Bangladeshi population to improve the differentiation between βTT and anemia (Table 1).

Analysis of individual CBC parameters (Table 2 and S1Table) showed that RBC had the best performance (AUC = 0.98; 95% CI: 0.96–0.99; p < 0.0001, DOR = 260; 95% CI: 131.27–702.91), clearly outperforming MCV and MCH.

**Table 2:** Sensitivity, specificity, and Youden’s Index, ROC-AUC, accuracy, and DOR of CBC parameters and ML models fitted to CBC parameters with their 95% confidence interval.

Feature/ Model	Sensitivity	Specificity	Youden’s Index	ROC-AUC	Accuracy	DOR
RBC	0.91 (0.85–0.95)	0.96 (0.94–0.98)	0.87 (0.82–0.92)	0.98 (0.96–0.99)	0.95 (0.92–0.96)	259.99 (126.48–749.83)
HGB	0.42 (0.34–0.50)	0.99 (0.98–1.00)	0.41 (0.33–0.49)	0.65 (0.59–0.72)	0.82 (0.78–0.85)	116.37 (35.90–855311.27)
MCH	0.92 (0.87–0.96)	0.88 (0.84–0.91)	0.8 (0.74–0.86)	0.94 (0.04–0.08)	0.89 (0.86–0.92)	99.97 (62.10–416.93)
MCV	0.86 (0.79–0.91)	0.84 (0.80–0.88)	0.7 (0.63–0.77)	0.91 (0.06–0.12)	0.85 (0.81–0.88)	34.93 (23.85–81.39)
RDW-CV	0.92 (0.87–0.96)	0.6 (0.55–0.66)	0.53 (0.46–0.60)	0.81 (0.76–0.84)	0.7 (0.66–0.74)	18.37 (9.54–52.03)
HCT	0.62 (0.54–0.70)	0.87 (0.83–0.90)	0.49 (0.40–0.58)	0.75 (0.69–0.81)	0.79 (0.75–0.83)	10.77 (7.27–24.75)
MCHC	0.6 (0.52–0.68)	0.78 (0.73–0.82)	0.38 (0.29–0.47)	0.67 (0.27–0.39)	0.73 (0.68–0.76)	5.77 (4.04–9.26)
LGBM	0.98 (0.94–0.99)	0.93 (0.90–0.95)	0.91 (0.88–0.95)	0.99 (0.98–0.99)	0.94 (0.92–0.96)	610.69 (258.51–11962369.86)
SVM	0.97 (0.93–0.99)	0.94 (0.91–0.96)	0.91 (0.88–0.95)	0.98 (0.97–0.99)	0.95 (0.92–0.97)	528.17 (282.55–13870375.84)
XGB	0.96 (0.91–0.98)	0.96 (0.93–0.97)	0.91 (0.87–0.95)	0.98 (0.97–0.99)	0.96 (0.93–0.97)	505.57 (231.39–2437.78)
RF	0.97 (0.93–0.99)	0.93 (0.90–0.95)	0.90 (0.87–0.94)	0.98 (0.97–0.99)	0.94 (0.92–0.96)	454.75 (196.44–2320.68)
ADA	0.94 (0.89–0.97)	0.96 (0.94–0.98)	0.91 (0.87–0.95)	0.98 (0.97–0.99)	0.96 (0.93–0.97)	438.73 (208.62–1888.72)
GB	0.97 (0.92–0.99)	0.94 (0.91–0.96)	0.90 (0.86–0.94)	0.97 (0.95–0.99)	0.95 (0.92–0.96)	419.5 (200.53–2227.31)
LR	0.97 (0.92–0.99)	0.92 (0.89–0.94)	0.88 (0.85–0.93)	0.98 (0.97–0.99)	0.93 (0.91–0.95)	316.33 (161.60–1495.00)
LDA	0.95 (0.90–0.98)	0.94 (0.91–0.96)	0.89 (0.85–0.93)	0.98 (0.96–0.99)	0.94 (0.92–0.96)	295.3 (147.05–1179.72)
KNN	0.97 (0.92–0.99)	0.91 (0.88–0.94)	0.88 (0.84–0.92)	0.97 (0.96–0.99)	0.93 (0.90–0.95)	291.76 (138.17–1392.07)
MLP	0.93 (0.88–0.96)	0.95 (0.93–0.97)	0.88 (0.84–0.93)	0.98 (0.97–0.99)	0.95 (0.92–0.96)	273.97 (150.63–973.89)
NB	0.97 (0.93–0.99)	0.88 (0.84–0.91)	0.85 (0.81–0.90)	0.97 (0.95–0.98)	0.91 (0.88–0.93)	246.71 (109.31–5311989.88)
DT	0.92 (0.86–0.95)	0.94 (0.91–0.96)	0.86 (0.80–0.91)	0.93 (0.90–0.95)	0.93 (0.91–0.95)	175.24 (92.64–435.99)

Then, machine learning models trained on CBC parameters demonstrated remarkable diagnostic performance for βTT detection using 7 CBC parameters (Table 2). XGB achieved the highest accuracy and specificity, with the best PPV, LR + , and diagnostic odds ratio (505.57, 95% CI: 231.39–2437.78), demonstrating its strong ability for confirming βTT cases. In our dataset including 143 βTT and 324 anemia cases, RBC misclassified 13 βTT and 12 anemia cases, while XGB misclassified 6 βTT and 14 anemia cases, indicating only modest gains of XGB over RBC for βTT classification.

Table 3 and S2 Table summarize the diagnostic metrics of discriminant formulas and ML models fitted to discriminant formulas. Two new formulas, DF-6 and DF-27, demonstrated reliable diagnostic performance.

**Table 3:** Sensitivity, specificity, and Youden’s Index, ROC-AUC, accuracy, and DOR of discriminant formulas and ML models fitted to discriminant formulas with their 95% confidence interval.

Feature/ Model	Sensitivity	Specificity	Youden’s Index	ROC-AUC	Accuracy	DOR
DF-06	0.94 (0.88–0.97)	0.96 (0.93–0.97)	0.89 (0.85–0.94)	0.98 (0.97–0.99)	0.95 (0.93–0.97)	329.68 (161.21–1025.13)
RBC	0.91 (0.85–0.95)	0.96 (0.94–0.98)	0.87 (0.82–0.92)	0.98 (0.96–0.99)	0.95 (0.92–0.96)	260 (131.27–702.91)
Srivastava	0.94 (0.89–0.97)	0.93 (0.90–0.95)	0.87 (0.82–0.92)	0.98 (0.96–0.99)	0.93 (0.91–0.95)	220.84 (110.56–668.12)
Ravanbakhsh -F1	0.89 (0.83–0.93)	0.96 (0.94–0.98)	0.85 (0.79–0.90)	0.97 (0.96–0.98)	0.94 (0.91–0.96)	206.37 (105.30–541.89)
Kerman I	0.97 (0.92–0.99)	0.88 (0.84–0.91)	0.84 (0.80–0.89)	0.97 (0.95–0.98)	0.91 (0.88–0.93)	201.69 (93.62–1031.90)
Hisham	0.94 (0.89–0.97)	0.92 (0.89–0.94)	0.86 (0.82–0.91)	0.97 (0.96–0.98)	0.93 (0.90–0.95)	193.41 (99.33–588.69)
Index 26	0.94 (0.89–0.97)	0.92 (0.88–0.94)	0.86 (0.81–0.91)	0.97 (0.96–0.99)	0.93 (0.90–0.95)	185.62 (94.32–645.97)
Alparslan	0.93 (0.88–0.96)	0.93 (0.90–0.95)	0.86 (0.81–0.91)	0.96 (0.94–0.98)	0.93 (0.90–0.95)	182.57 (91.25–497.26)
Janel (11T)	0.9 (0.84–0.94)	0.95 (0.92–0.97)	0.85 (0.80–0.91)	0.96 (0.96–0.99)	0.94 (0.91–0.95)	177.37 (93.96–455.33)
Mentzer	0.96 (0.91–0.98)	0.88 (0.84–0.91)	0.84 (0.79–0.88)	0.97 (0.96–0.98)	0.9 (0.87–0.93)	166.86 (82.09–577.66)
DF27	0.93 (0.88–0.96)	0.92 (0.89–0.95)	0.85 (0.80–0.90)	0.97 (0.96–0.99)	0.93 (0.90–0.95)	159.07 (81.47–432.20)
Ehsani	0.96 (0.91–0.98)	0.87 (0.83–0.90)	0.83 (0.78–0.87)	0.97 (0.95–0.98)	0.90 (0.86–0.92)	149.21 (72.80–599.23)
Merdin-2	0.94 (0.88–0.97)	0.87 (0.83–0.90)	0.81 (0.75–0.86)	0.96 (0.94–0.98)	0.89 (0.86–0.92)	99.97 (52.84–264.25)
Sehgal	0.92 (0.87–0.96)	0.87 (0.85–0.92)	0.81 (0.75–0.87)	0.96 (0.94–0.98)	0.90 (0.87–0.92)	96 (51.42–242.72)
Jayabose	0.92 (0.86–0.95)	0.87 (0.85–0.92)	0.81 (0.75–0.86)	0.95 (0.92–0.97)	0.90 (0.87–0.92)	90.14 (49.59–207.30)
Hameed	0.92 (0.86–0.95)	0.87 (0.85–0.92)	0.81 (0.75–0.86)	0.95 (0.92–0.97)	0.9 (0.87–0.92)	90.14 (49.59–207.30)
Kerman II	0.92 (0.86–0.95)	0.87 (0.85–0.92)	0.8 (0.74–0.86)	0.96 (0.94–0.97)	0.9 (0.87–0.92)	87.33 (46.96–212.12)
Merdin-1	0.90 (0.84–0.94)	0.87 (0.85–0.92)	0.79 (0.73–0.85)	0.95 (0.94–0.97)	0.9 (0.8640–0.9197)	76.08 (42.60–167.78)
Das Gupta	0.80 (0.72–0.85)	0.95 (0.92–0.97)	0.75 (0.68–0.81)	0.92 (0.89–0.95)	0.9 (0.86–0.92)	75.67 (42.97–153.72)
Bordbar	0.92(0.87–0.96)	0.86 (0.82–0.89)	0.78 (0.72–0.84)	0.92 (0.89–0.95)	0.88 (0.85–0.91)	74.4 (40.02–176.05)
Keikhaei	0.90 (0.84–0.94)	0.89 (0.85–0.92)	0.79 (0.73–0.85)	0.94 (0.92–0.97)	0.89 (0.86–0.92)	73.71 (41.14–160.91)
England and Fraser (E&F)	0.90 (0.84–0.94)	0.88 (0.84–0.91)	0.78 (0.72–0.84)	0.95 (0.92–0.97)	0.89 (0.86–0.91)	69.35 (38.40–153.26)
Shine and Lal (S&L)	0.90 (0.83–0.94)	0.88 (0.84–0.91)	0.77 (0.71–0.83)	0.93 (0.90–0.95)	0.88 (0.85–0.91)	62.36 (35.20–129.33)
Sirdah	0.87 (0.80–0.91)	0.9 (0.87–0.93)	0.77 (0.70–0.84)	0.95 (0.93–0.97)	0.89 (0.86–0.92)	61.68 (34.72–129.38)
TI (MDHL)	0.88 (0.82–0.92)	0.89 (0.85–0.92)	0.77 (0.71–0.83)	0.93 (0.90–0.96)	0.89 (0.86–0.91)	61.2 (34.54–125.66)
SCS_BTT_	0.91 (0.85–0.95)	0.85 (0.80–0.88)	0.75 (0.69–0.81)	0.93 (0.90–0.95)	0.87 (0.83–0.89)	54.8 (30.83–118.29)
Wongprachum	0.89 (0.83–0.93)	0.86 (0.82–0.89)	0.75 (0.69–0.81)	0.94 (0.92–0.96)	0.87 (0.84–0.90)	49.21 (29.18–102.46)
Nishad (Thal)	0.9 (0.83–0.94)	0.85 (0.81–0.89)	0.75 (0.68–0.81)	0.92 (0.89–0.95)	0.87 (0.83–0.89)	49.07 (27.74–105.30)
Ravanbakhsh-F4	0.88 (0.82–0.92)	0.87 (0.83–0.90)	0.75 (0.68–0.81)	0.92 (0.90–0.95)	0.87 (0.84–0.90)	48.44 (28.37–99.14)
Roth	0.87 (0.80–0.91)	0.84 (0.80–0.88)	0.71 (0.64–0.78)	0.91 (0.88–0.94)	0.85 (0.81–0.88)	34.93 (20.58–66.81)
Green and King (G&K)	0.73 (0.66–0.80)	0.92 (0.89–0.94)	0.65 (0.57–0.73)	0.89 (0.85–0.92)	0.86 (0.83–0.89)	31.67 (18.92–57.45)
Kandhro-1	0.94 (0.88–0.97)	0.6 (0.54–0.65)	0.54 (0.47–0.60)	0.81 (0.77–0.85)	0.7 (0.66–0.74)	22.22 (12.07–59.59)
Sirachainan	0.59 (0.51–0.67)	0.94 (0.90–0.96)	0.53 (0.45–0.61)	0.77 (0.71–0.82)	0.83 (0.79–0.86)	21.15 (12.55–39.89)
Ravanbakhsh-F3	0.75 (0.67–0.81)	0.88 (0.84–0.91)	0.62 (0.54–0.70)	0.88 (0.85–0.92)	0.84 (0.80–0.87)	21.1 (12.94–36.58)
Bessman	0.92 (0.87–0.96)	0.60 (0.55–0.65)	0.52 (0.45–0.59)	0.8 (0.76–0.84)	0.7 (0.66–0.74)	18.14 (10.42–44.54)
Kandhro-2	0.66 (0.57–0.73)	0.90 (0.86–0.92)	0.56 (0.47–0.64)	0.83 (0.79–0.87)	0.83 (0.79–0.86)	17.51 (10.93–29.86)
Ricerca	0.66 (0.57–0.73)	0.90 (0.86–0.92)	0.56 (0.47–0.64)	0.83 (0.79–0.87)	0.83 (0.79–0.86)	17.51 (10.93–29.86)
Plengsuree	0.66 (0.58–0.73)	0.90 (0.86–0.92)	0.56 (0.47–0.64)	0.83 (0.79–0.87)	0.83 (0.79–0.86)	17.51 (10.93–29.86)
Matos and Carvalho (M&C)	0.76 (0.68–0.82)	0.85 (0.81–0.88)	0.6 (0.52–0.68)	0.87 (0.82–0.90)	0.82 (0.78–0.85)	17.32 (10.91–29.75)
Zaghloul-1	0.66 (0.58–0.74)	0.90 (0.86–0.92)	0.56 (0.47–0.64)	0.79 (0.74–0.84)	0.82 (0.79–0.86)	16.88 (10.59–28.96)
Ravanbakhsh-F2	0.65 (0.57–0.72)	0.88 (0.84–0.91)	0.53 (0.44–0.61)	0.79 (0.74–0.84)	0.81 (0.77–0.84)	13.59 (8.62–22.29)
Sargolzaie	0.26 (0.19–0.34)	0.96 (0.94–0.98)	0.22 (0.15–0.30)	0.61 (0.55–0.67)	0.75 (0.71–0.78)	9.08 (4.8–21.02)
Zaghloul-2	0.56 (0.48–0.64)	0.86 (0.82–0.89)	0.42 (0.33–0.51)	0.7 (0.64–0.76)	0.77 (0.73–0.80)	7.67 (4.93–12.63)
TI (MCHD)	0.64 (0.56–0.72)	0.77 (0.73–0.82)	0.42 (0.33–0.51)	0.68 (0.62–0.74)	0.73 (0.69–0.77)	6.2 (4.09–9.70)
Pornprasert	0.62 (0.54–0.70)	0.78 (0.73–0.82)	0.4 (0.31–0.49)	0.67 (0.61–0.73)	0.73 (0.69–0.77)	5.77 (3.87–9.10)
Cruise	0.25 (0.19–0.33)	0.94 (0.91–0.96)	0.19 (0.11–0.27)	0.54 (0.48–0.60)	0.73 (0.69–0.77)	5.11 (2.79–9.97)
Huber-Herklotz	0.75 (0.67–0.81)	0.45 (0.40–0.51)	0.20 (0.20–0.29)	0.61 (0.56–0.67)	0.54 (0.50–0.59)	2.47 (1.63–3.93)
SVM	0.97 (0.92–0.99)	0.94 (0.91–0.96)	0.90 (0.87–0.95)	0.97 (0.95–0.99)	0.95 (0.92–0.96)	419.52 (206.56–2180.43)
ADA	0.97 (0.93–0.99)	0.92 (0.88–0.94)	0.89 (0.86–0.93)	0.98 (0.97–0.99)	0.93 (0.91–0.95)	382.25 (164.87–1729.01)
GB	0.97 (0.93–0.99)	0.91 (0.88–0.94)	0.89 (0.86–0.93)	0.98 (0.97–0.99)	0.93 (0.90–0.95)	367.36 (180.53–1839.04)
LR	0.97 (0.92–0.99)	0.93 (0.90–0.95)	0.89 (0.86–0.94)	0.98 (0.97–0.99)	0.94 (0.91–0.96)	361.20 (179.81–1971.20)
RF	0.94 (0.88–0.97)	0.96 (0.93–0.98)	0.90 (0.87–0.94)	0.98 (0.97–0.99)	0.95 (0.93–0.97)	329.68 (183.04–1734.07)
XGB	0.95 (0.90–0.98)	0.95 (0.92–0.97)	0.90 (0.86–0.94)	0.98 (0.97–0.99)	0.95 (0.92–0.97)	350.86 (180.26–1488.11)
MLP	0.96 (0.91–0.98)	0.95 (0.90–0.96)	0.89 (0.86–0.94)	0.98 (0.96–0.99)	0.94 (0.92–0.96)	378.87 (195.83–2302.12)
LGBM	0.94 (0.89–0.97)	0.95 (0.92–0.97)	0.89 (0.86–0.94)	0.98 (0.97–0.99)	0.95 (0.92–0.96)	304.74 (169.56–1323.47)
LDA	0.93 (0.88–0.96)	0.95 (0.93–0.97)	0.88 (0.85–0.93)	0.95 (0.92–0.98)	0.95 (0.92–0.96)	273.98 (143.49–919.86)
NB	0.96 (0.91–0.98)	0.91 (0.88–0.94)	0.87 (0.83–0.92)	0.97 (0.96–0.98)	0.93 (0.90–0.95)	241.38 (119.96–915.18)
KNN	0.96 (0.91–0.98)	0.91 (0.87–0.94)	0.87 (0.83–0.92)	0.97 (0.95–0.98)	0.93 (0.90–0.95)	232.27 (122.83–772.17)
DT	0.86 (0.79–0.91)	0.94 (0.91–0.96)	0.80 (0.74–0.86)	0.90 (0.87–0.93)	0.92 (0.89–0.94)	79.59 (45.63–169.25)

**Fig 7:**
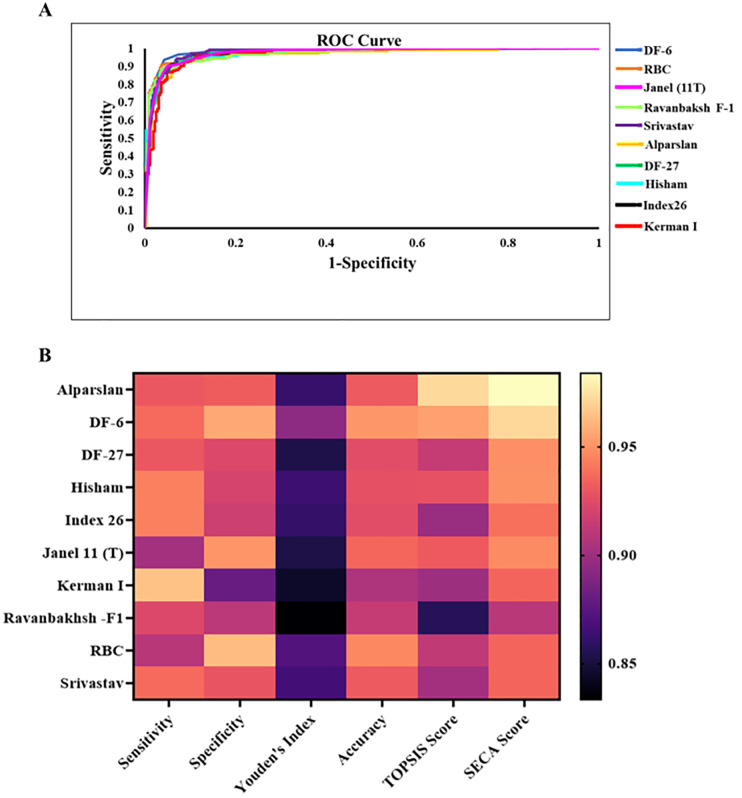
Graphical overview of the performance of the top ten discriminant formulas. **(A)** ROC curves for each discriminant formula, **(B)** Comparison of sensitivity, specificity, Youden’s Index, accuracy, TOPSIS score, and SECA score across the top ten discriminant formulas.

Among the existing formulas, Kerman I displayed the highest sensitivity, NPV, and the lowest FNR. RBC achieved the highest specificity and the lowest FPR. DF-6 exhibited the highest Youden’s index at its optimal cut-off of ≥4. Overall, DF-6 exhibited the best diagnostic performance across accuracy, DOR (329.68, 95% CI: 161.21–1025.13) at a cut-off of ≥4. Notably, the highest ROC-AUC value was achieved by both DF-6 (0.98, 95% CI: 0.97–0.99, p < 0.001) and RBC, confirming their strong diagnostic performance (Fig 7A).

Assessment of ML models trained on 47 discriminant formulas constantly exhibited high performance for the detection of βTT cases. LGBM attained the highest ROC-AUC and demonstrated high sensitivity and specificity. XGB exhibited the highest sensitivity and NPV. SVM demonstrated superior discriminative performance with the highest accuracy and DOR (419.52, 95% CI: 206.56–2180.43), achieving both sensitivity and specificity above 95%.

In the dataset, DF-6 misclassified 9 βTT and 14 anemia cases, while SVM misclassified 5 βTT and 20 anemia cases, indicating only modest gains of SVM over DF-6 for βTT classification.

### 3.3. Ranking by multi-criteria decision making (MCDM) analysis

#### 3.3.1. MCDM analysis on CBC parameters and discrimination formulas.

In MCDM analysis of CBC parameters and discrimination indices, DF-06 ranked first across TOPSIS and SECA methods, obtaining the highest scores, and highlighting its strong diagnostic performance. RBC ranked second with a high TOPSIS and SECA scores. Janel (11T), Ravanbakhsh-F1, Srivastava, Alparslan, DF27, Hisham, Index 26, Kerman I ranked within the top ten, indicating their reliable performance (Fig 7B) and (Table 4).

**Table 4:** MCDM ranking of CBC parameters and discriminant formulas.

Indices	TOPSIS Score	TOPSIS Rank	SECA Score	SECA Rank
DF-06	0.9675	1	0.9805	1
RBC	0.948	2	0.9687	2
Ravanbakhsh -F1	0.9279	3	0.9511	3
Janel (11T)	0.9263	4	0.9443	5
Srivastava	0.9156	5	0.9502	4
Alparslan	0.9137	6	0.9371	6
Hisham	0.9016	7	0.9369	7
DF27	0.9007	8	0.932	9
Index 26	0.8975	9	0.9352	8
Kerman I	0.8582	10	0.9111	10
Mentzer	0.8569	11	0.9072	11
Sehgal	0.8549	12	0.8847	13
Hameed	0.8543	13	0.8783	15
Jayabose	0.8543	14	0.8782	16
Kerman II	0.8517	15	0.8785	14
Merdin-1	0.8479	16	0.8696	18
Ehsani	0.8435	17	0.8935	12
Keikhaei	0.8432	18	0.8614	19
Merdin-2	0.8402	19	0.8776	17
Sirdah	0.8398	20	0.8549	21
England and Fraser (E&F)	0.8375	21	0.8585	20
TI (MDHL)	0.8337	22	0.8454	23
Shine and Lal (S&L)	0.8282	23	0.8417	25
Das Gupta	0.8271	24	0.8459	22
Bordbar	0.8211	25	0.8421	24
Wongprachum	0.8073	26	0.8245	26
Ravanbakhsh-F4	0.8067	27	0.8153	28
SCS_BTT_	0.7999	28	0.8179	27
Nishad (Thal)	0.799	29	0.8119	29
Roth	0.773	30	0.7763	30
Green and King (G&K)	0.7422	31	0.7491	31
MCH	0.7412	32	0.7412	32
Ravanbakhsh-F3	0.7166	33	0.7093	33
Matos and Carvalho	0.6952	34	0.6788	34
MCV	0.6646	35	0.6646	35
Kandhro-2	0.6558	36	0.6463	36
Ricerca	0.6558	37	0.6463	37
Plengsuree	0.6558	38	0.6463	38
Zaghloul-1	0.6475	39	0.6258	39
Ravanbakhsh-F2	0.6258	40	0.6008	40
Sirachainan	0.6216	41	0.6118	41
Kandhro-1	0.5874	42	0.582	42
Bessman	0.5822	43	0.5697	43
Zaghloul-2	0.5221	44	0.4674	44
TI (MCHD)	0.5098	45	0.4399	46
Pornprasert	0.4954	46	0.4233	48
HGB	0.4574	47	0.4574	45
Sargolzaie	0.4359	48	0.3402	49
HCT	0.4345	49	0.4345	47
Cruise	0.4054	50	0.2624	50
Huber- Herklotz	0.3669	51	0.2105	51

#### 3.3.2. MCDM analysis on ML models.

The performance of ML models across CBC parameters was evaluated by MCDM analysis. According to the TOPSIS and SECA scores, XGB constantly attained the highest overall performance, followed by SVM, LGBM, and GBM. The assessment of ML models across 47 discriminant formulas revealed that SVM emerged as the top-performing model for βTT detection, followed by LR, XGB, GBM (Table 5).

**Table 5:** MCDM ranking of machine learning models for CBC parameters and discriminant formulas.

Model	TOPSIS Score		TOPSIS Rank		SECA Score		SECA Rank	
	CBC	Formula	CBC	Formula	CBC	Formulas	CBC	Formula
XGBoost (XGB)	0.79	0.765	1	3	0.79	0.765	1	3
LightGBM (LGBM)	0.768	0.709	3	7	0.768	0.709	3	7
Support Vector Machine	0.779	0.853	2	1	0.779	0.853	2	1
ADABoost (ADA)	0.595	0.742	10	5	0.595	0.742	10	5
Logistics Regression (LR)	0.674	0.797	6	2	0.674	0.797	6	2
Multi-layer perceptron (MLP)	0.598	0.714	9	6	0.598	0.714	9	6
Random Forest (RF)	0.748	0.663	5	10	0.748	0.663	5	10
Gradient Boosting (GBM)	0.759	0.755	4	4	0.759	0.755	4	4
Linear Discriminant Analysis (LDA)	0.662	0.604	7	11	0.662	0.604	7	11
K-Nearest Neighbours (KNN)	0.648	0.683	8	9	0.648	0.683	8	9
Gaussian Naive Bayes (GNB)	0.556	0.695	11	8	0.556	0.695	11	8
Decision Tree (DT)	0.336	0.112	12	12	0.336	0.112	12	12

### 3.4. Hierarchical clustering

The hierarchical clustering dendrogram was constructed using Ward’s linkage method with Euclidean distance. The number of clusters was determined, the dendrogram was examined at a Ward’s linkage distance threshold of approximately 5.0. At this threshold, CBC parameters and discriminant indices were classified into two main groups for their diagnostic performance.

**Fig 8:**
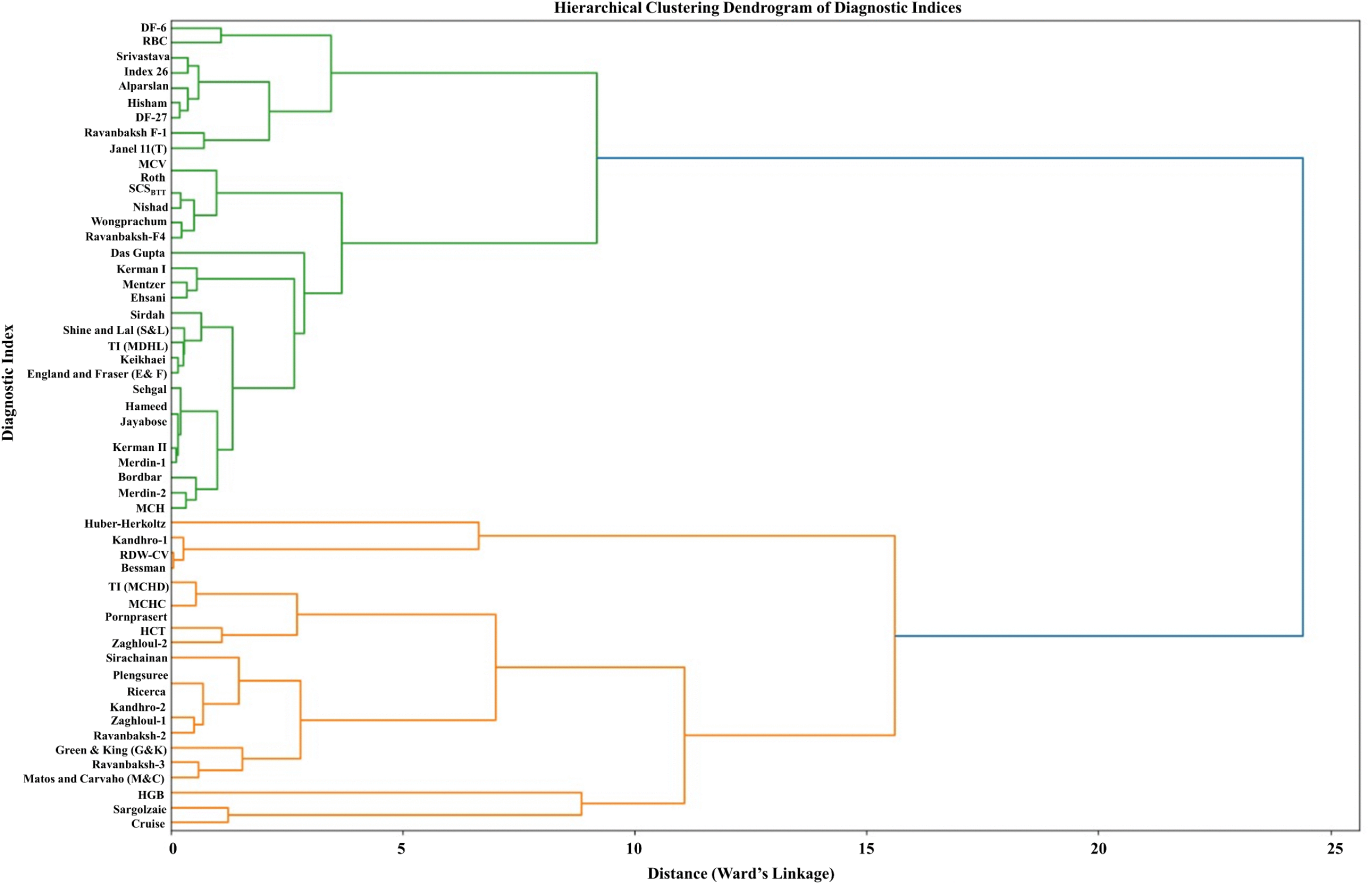
Hierarchical clustering dendrogram for CBC parameters and discriminant formulas, including homogeneous groups with similar diagnostic performance.

Group 1 included strong to moderate performing indices such as DF-06, RBC, Ravanbakhsh-F1, Srivastav, Janel (11T), Alparslan, Hisham, Index 26, DF-27, and Kerman I which (<2.5) formed tighter subclusters at lower linkage distances (<2.5). Conversely, group 2 comprised weak-performing indices such as Cruise, Huber–Herklotz, Pornprasert, Sargolzaie, and TI (MCHD), which clustered at higher linkage distances (>5.0). Their performance profiles were significantly different from the more reliable indices (Fig 8), which was also reflected by lower MCDM rankings. There were two major clusters in the dendrogram of ML models for CBC parameters at a linkage distance threshold of approximately 3.0. Cluster 1 included DT, GNB, MLP, KNN, LR, and LDA which demonstrated moderate diagnostic similarity. Cluster 2 included ADA, RF, GBM, LGBM, and SVM, and clustered at a lower linkage distance and showed high similarities in diagnostic metrics. XGB formed a separate sub-cluster between the two clusters due to its slightly different performance metrics, reflecting the highest overall performance.

**Fig 9:**
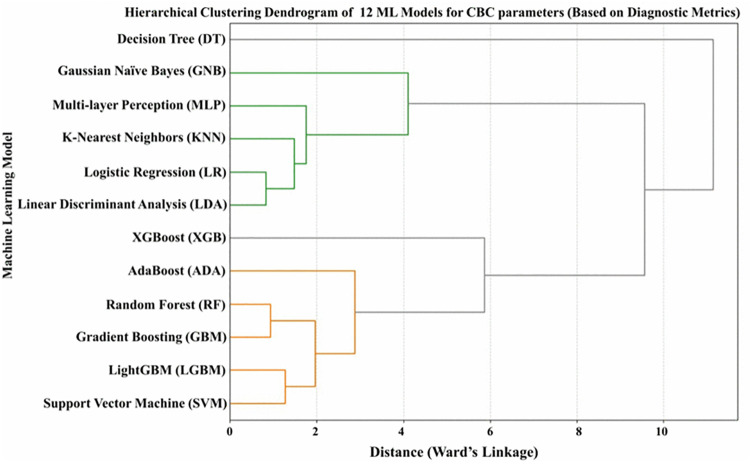
Hierarchical clustering dendrogram of ML models for CBC parameters, including homogeneous groups with similar diagnostic performance.

**Fig 10:**
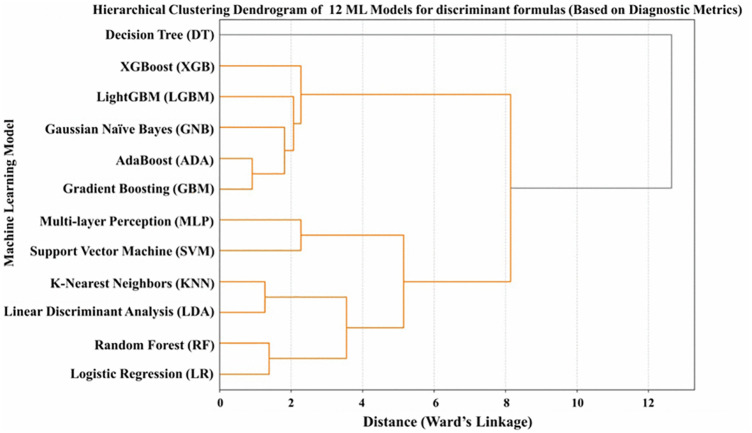
Hierarchical clustering dendrogram of ML models for discriminant formulas, including homogeneous groups with similar diagnostic performance.

Also, for 47 formulas, clustering analysis of ML models displayed two distinct clusters. The first cluster included LR, RF, LDA, K-NN, SVM, and MLP, which exposed strong similarity in their diagnostic metrics. The second cluster included GBM, LGBM, ADA, XGB, GNB, and DT. Among these, the ensemble boosting methods: GBM, LGBM, ADA, and XGB formed a closely linked subcluster, reflecting their consistent diagnostic outcome. In contrast, DT formed a different branch at the highest linkage distance, indicating comparatively weaker performance (Figs 9 and 10).

## 4. Discussion

The study illustrates a pathway that provides more accurate, and population-tailored diagnostic approaches for large scale screening of βTT and anemia in Bangladesh. The novelty of this work lies in introduction of DF-6 and DF-27 as optimized formulas and integration of 12 ML models to capture nonlinear relationships within hematological data. The newly developed composite index, DF-6 and ML models such as XGB and SVM can distinguish βTT from anemia in a Bangladeshi cohort with AUCs around 0.97–0.98, outperforming most established indices. Among individual hematological parameters, RBC was the most reliable CBC-based predictor of βTT, which is consistent with previous studies [18]. However, DF-6 and ML models (XGB and SVM) offered optimal balance between sensitivity and specificity. The genetic defects in β-globin synthesis trigger ineffective erythropoiesis in βTT while anemia is associated with nutritional deficiencies. DF-6 integrates combination of formulas with major CBC parameters that reflect the significant physiological differences between βTT and anemia. RBC, HGB, HCT, and RDW-CV were lower while MCV, MCH, and MCHC were higher in the anemic group than in the βTT group. High RBC count reflects ineffective erythropoiesis, while reduced MCV and MCH indicate microcytosis and hypochromia in βTT. Therefore, biological relevant parameters influenced distinct pathophysiological patterns of βTT and anemia, which explains superior discriminatory performance of DF-6. Several previously proposed indices such as S & L, SCS_BTT_, Roth, Index26, and Cruise indices demonstrated strong performance in different populations [12–14,17]. However, in this study, the combination of formulas provided better diagnostic performance than individual formulas. The top-performing formulas (DF-6, RBC, Janel (11T), Ravanbakhsh-F1, Srivastava, Alparslan, DF27, Hisham, Index 26, and Kerman I) demonstrated better and more balanced outcomes compared to some other studies [12,13,17]. Kandhro et.al previously reported RBC, RDW, S&L, EF, Srivastav, Sirdah, G&K, RDWI, Huber–Herklotz, Kerman1, Kandhro1, Kandhro2 indices obtained 100% sensitivity and specificity [53]. However, no other study could reproduce these results [4,10–12,60–63]. In contrast, the present study observed diminished performance for Kandhro-1, Kandhro-2, and Ricerca, while indices such as Cruise, Huber-Herklotz, Pornprasert, and Sargolzaie showed limited diagnostic utility in the Bangladeshi population. These findings highlighted that the influence of population-specific hematological profiles on the discriminatory power of the widely used indices. Genetic heterogeneity increases complexity as different communities inherit different β-thalassemia mutations, each with variable effects on hematological parameters. Some mutations lead to easily identifiable changes in red cell indices, while others result in milder or atypical patterns, restraining the consistency of some discriminant formulas [4,10,11,60–63]. Additionally, coexisting conditions, including iron deficiency, α-thalassemia, E/β-thalassemia, hemoglobin S/β-thalassemia and deficiencies in vitamin B12 or folate, can further alter typical hematological parameters, reducing the effectiveness of conventional screening tools. Smaller, homogeneous study cohorts may report higher efficacy of standard indices, but larger and more diverse datasets tend to reveal their limitations [12,44,53]. These are the common challenges reported in the population of many countries, including Pakistan [53,64], India [65], Iran [66], Turkey [67] and Saudi Arabia [68]. In South Asia, extreme heterogeneity, has been reported throughout different parts of India [13]. Pakistan also reflected significant genetic diversity due to migrations, invasions, and commercial interactions [53]. Conversely, in recent years, ML models enhanced the detection of βTT by capturing complex, nonlinear relationships within data. The strong diagnostic performance of XGB and SVM is consistent with previous reports [7,20,69]. Also, in some recent studies [19–21], boosting algorithms (ADA, GB, and XGB) performed well in the detection of thalassemia. Therefore, integration of ML models with population-tailored discriminant formulas may play a significant role to improve diagnostic accuracy for inherited and nutritional anemias in large populations [12,44].

In Bangladesh, DF-6 ≥ 4 could be prioritized for confirmatory hemoglobin electrophoresis in premarital or antenatal screening programs. While, DF-6 < 4 could be prioritized for monitoring iron profile. Similarly, ML models, XGB, SVM could be integrated into laboratory software systems to enable rapid and cost-effective screening in resource-limited settings. These approaches exert significant potential to alleviate the healthcare burden posed by hemoglobinopathies, particularly in low- and middle-income countries where access to comprehensive screening is limited [12,17].

### 4.1. Limitations

Certain limitations of the study should be acknowledged. The study sample were collected from university and tertiary care settings, which may not represent rural primary-care populations. In addition, CBC and hemoglobin electrophoresis were used as the primary confirmatory test. Incorporating iron profile and molecular confirmation would further improve diagnostic performance of discriminant formulas and ML models by reducing the risk of misclassification. Furthermore, the cut-off values for discriminant formulas were optimized specifically for the Bangladeshi population. Therefore, the applicability of the formulas and ML models to other South Asian populations or global cohorts requires external validation. Despite the advancements, some intrinsic challenges and areas for future research are vital for the evolution and clinical implementation of these technologies. To accelerate broader acceptance, future research should emphasize improving the interpretability of ML models using SHapley Additive exPlanations (SHAP) and Local Interpretable Model Agnostic Explanation (LIME). Additionally, user-friendly interfaces should be developed that enable their integration into routine clinical practice [17].

## 5. Conclusion

The study demonstrated that population specific discriminant formulas, DF-6 and advanced machine learning models, XGB and SVM accurately distinguish β-thalassemia trait from other anemias within the Bangladeshi population. The results emphasized the need for population-specific optimization of cut-off values for the formulas. High-performing CBC-based discriminant formulas and powerful ML models enhanced the diagnostic accuracy of βTT detection by addressing the genetic diversity and diagnostic challenges. Notably, the application of proposed XGB and SVM models require minimal computational facility. It can perform analysis on standard laboratory computers without specialized hardware. Therefore, this approach can be feasible for integration into routine diagnostic facilities of laboratories in Bangladesh. These cost-effective, scalable approaches can significantly improve early detection and management of β-thalassemia carriers in resource-limited settings, ultimately optimizing healthcare resources in low- and middle-income countries.

## Supporting information

S1 Table True positive (TP), true negative (TN), false positive (FP), and false negative (FN), false positive rate (FPR), false negative rate (FNR), positive predictive value (PPV), negative predictive value (NPV), positive likelihood ratio (LR+), negative likelihood ratio (LR-) of CBC parameters and ML models fitted to CBC parameters with their 95% confidence interval. (DOCX)

S2 Table True positive (TP), true negative (TN), false positive (FP), and false negative (FN), false positive rate (FPR), false negative rate (FNR), positive predictive value (PPV), negative predictive value (NPV), positive likelihood ratio (LR+), negative likelihood ratio (LR-) of discriminant formulas and ML models fitted to discriminant formulas with their 95% confidence interval. (DOCX)
