# Supplementary material for: Diagnostic performance of discriminant formulas and machine learning models for detecting β-thalassemia trait in Bangladesh
Source: PLoS One. 2026 Jun 16;21(6):e0350387. doi: 10.1371/journal.pone.0350387 (PMC13271480; doi:10.1371/journal.pone.0350387)
Supplement: S2 Table — (DOCX) [file pone.0350387.s002.docx]

**Table S2. True positive (TP), true negative (TN), false positive (FP), and false negative (FN), false positive rate (FPR), false negative rate (FNR), positive predictive value (PPV), negative predictive value (NPV), positive likelihood ratio (LR+), negative likelihood ratio (LR-) of discriminant formulas and ML models fitted to discriminant formulas with their 95% confidence interval.**

| **Feature/**  **Model** | **TP** | **TN** | **FP** | **FN** | **FPR** | **FNR** | **PPV** | **NPV** | **LR+** | **LR-** |
| --- | --- | --- | --- | --- | --- | --- | --- | --- | --- | --- |
| DF-06 | 134 | 310 | 14 | 9 | 0.04 (0.03-0.07) | 0.06 (0.03-0.12) | 0.91 (0.85-0.94) | 0.97 (0.95-0.99) | 21.69 (14.02-40.94) | 0.07 (0.03-0.11) |
| RBC | 130 | 312 | 12 | 13 | 0.04 (0.02-0.06) | 0.09 (0.05-0.15) | 0.92 (0.86-0.95) | 0.96 (0.93-0.98) | 24.55 (15.39-49.67) | 0.09 (0.05-0.14) |
| Srivastav | 135 | 301 | 23 | 8 | 0.07 (0.05-0.10) | 0.06 (0.03-0.11) | 0.85 (0.79-0.90) | 0.97 (0.95-0.99) | 13.30 (9.26-20.85) | 0.06 (0.02-0.10) |
| Ravanbakhsh -F1 | 127 | 312 | 12 | 16 | 0.04 (0.02-0.06) | 0.11 (0.07-0.17) | 0.91 (0.86-0.95) | 0.95 (0.92-0.97) | 23.98 (15.06-48.34) | 0.12 (0.07-0.17) |
| Kerman I | 138 | 285 | 39 | 5 | 0.12 (0.09-0.16) | 0.03 (0.02-0.08) | 0.78 (0.71-0.83) | 0.98 (0.96-0.99) | 8.02 (6.14-11.20) | 0.04 (0.01-0.08) |
| Hisham | 135 | 298 | 26 | 8 | 0.08 (0.06-0.12) | 0.06 (0.03-0.11) | 0.84 (0.77-0.89) | 0.97 (0.95-0.99) | 11.76 (8.54-17.68) | 0.06 (0.02-0.10) |
| Index 26 | 135 | 297 | 27 | 8 | 0.08 (0.06-0.12) | 0.06 (0.03-0.11) | 0.83 (0.77-0.88) | 0.97 (0.95-0.99) | 11.33 (8.25-17.17) | 0.06 (0.02-0.10) |
| Alparslan | 133 | 302 | 22 | 10 | 0.07 (0.05-0.10) | 0.07 (0.04-0.12) | 0.86 (0.79-0.90) | 0.97 (0.94-0.98) | 13.7 (9.42-22.48) | 0.08 (0.03-0.12) |
| Janel (11T) | 129 | 308 | 16 | 14 | 0.05 (0.03-0.08) | 0.10 (0.06-0.16) | 0.89 (0.83-0.93) | 0.96 (0.93-0.97) | 18.27 (12.28-32.92) | 0.10 (0.05-0.15) |
| Mentzer | 137 | 285 | 39 | 6 | 0.12 (0.09-0.16) | 0.04 (0.02-0.09) | 0.78 (0.71-0.83) | 0.98 (0.96-0.99) | 7.96 (6.17-11.15) | 0.05 (0.02-0.09) |
| DF27 | 133 | 299 | 25 | 10 | 0.08 (0.05-0.11) | 0.07 (0.04-0.12) | 0.84 (0.78-0.89) | 0.97 (0.94-0.98) | 12.05 (8.68-18.40) | 0.08 (0.03-0.12) |
| Ehsani | 137 | 281 | 43 | 6 | 0.13 (0.10-0.17) | 0.04 (0.02-0.09) | 0.76 (0.69-0.82) | 0.98 (0.96-0.99) | 7.22 (5.63-9.76) | 0.05 (0.02-0.09) |
| Merdin-2 | 134 | 282 | 42 | 9 | 0.13 (0.10-0.17) | 0.06 (0.03-0.12) | 0.76 (0.69-0.82) | 0.97 (0.94-0.98) | 7.23 (5.54-10.02) | 0.07 (0.03-0.12) |
| Sehgal | 132 | 288 | 36 | 11 | 0.11 (0.08-0.15) | 0.08 (0.04-0.13) | 0.79 (0.72-0.84) | 0.96 (0.94-0.98) | 8.31 (6.35-11.73) | 0.09 (0.04-0.14) |
| Jayabose | 131 | 289 | 35 | 12 | 0.11 (0.08-0.15) | 0.08 (0.05-0.14) | 0.79 (0.72-0.84) | 0.96 (0.93-0.98) | 8.48 (6.49-11.97) | 0.09 (0.05-0.15) |
| Hameed | 131 | 289 | 35 | 12 | 0.11 (0.08-0.15) | 0.08 (0.05-0.14) | 0.79 (0.72-0.84) | 0.96 (0.93-0.98) | 8.48 (6.49-11.97) | 0.09 (0.05-0.15) |
| Kerman II | 131 | 288 | 36 | 12 | 0.11 (0.08-0.15) | 0.08 (0.05-0.14) | 0.78 (0.72-0.84) | 0.96 (0.93-0.98) | 8.24 (6.31-11.64) | 0.09 (0.04.15) |
| Merdin-1 | 129 | 289 | 35 | 14 | 0.11 (0.08-0.15) | 0.10 (0.06-0.16) | 0.79 (0.72-0.84) | 0.95 (0.92-0.97) | 8.35 (6.24-12.07) | 0.11 (0.06-0.17) |
| Das Gupta | 114 | 308 | 16 | 29 | 0.05 (0.03-0.08) | 0.20 (0.15-0.28) | 0.88 (0.81-0.92) | 0.91 (0.88-0.94) | 16.14 (10.67-28.48) | 0.21 (0.15-0.28) |
| Bordbar | 132 | 279 | 45 | 11 | 0.14 (0.11-0.18 | 0.08 (0.04-0.13) | 0.75 (0.68-0.80) | 0.96 (0.93-0.98) | 6.65 (5.14-8.97) | 0.09 (0.04-0.14) |
| Keikhaei | 129 | 288 | 36 | 14 | 0.11 (0.08-0.15) | 0.10 (0.06-0.16) | 0.78 (0.71-0.84) | 0.95 (0.92-0.97) | 8.12 (6.18-11.41) | 0.11 (0.06-0.17) |
| England and Fraser (E&F) | 129 | 286 | 38 | 14 | 0.12 (0.09-0.16) | 0.10 (0.06-0.16) | 0.77 (0.70-0.83) | 0.95 (0.92-0.97) | 7.69 (5.77-10.86) | 0.11 (0.06-0.17) |
| Shine and Lal (S&L) | 128 | 285 | 39 | 15 | 0.12 (0.09-0.16) | 0.10 (0.06-0.17) | 0.77 (0.70-0.82) | 0.95 (0.92-0.97) | 7.44 (5.66-10.34) | 0.12 (0.06-0.18) |
| Sirdah | 124 | 293 | 31 | 19 | 0.10 (0.07-0.13) | 0.13 (0.09-0.20) | 0.8 (0.73-0.86) | 0.94 (0.91-0.96) | 9.06 (6.64-13.32) | 0.15 (0.09-0.21) |
| TI (MDHL) | 126 | 289 | 35 | 17 | 0.11 (0.08-0.15) | 0.12 (0.08-0.18) | 0.78 (0.71-0.84) | 0.94 (0.91-0.97) | 8.16 (6.06-11.75) | 0.13 (0.08-0.20) |
| SCSBTT | 130 | 274 | 50 | 13 | 0.15 (0.12-0.20) | 0.09 (0.05-0.15) | 0.72 (0.65-0.78) | 0.95 (0.92-0.97) | 5.89 (4.62-7.81) | 0.11 (0.06-0.17) |
| Wongprachum | 127 | 279 | 45 | 16 | 0.14 (0.11-0.18) | 0.11 (0.07-0.17) | 0.74 (0.67-0.80) | 0.95 (0.91-0.97) | 6.39 (5.03-8.69) | 0.13 (0.07-0.20) |
| Nishad (Thal) | 128 | 276 | 48 | 15 | 0.15 (0.11-0.19) | 0.10 (0.06-0.17) | 0.73 (0.66-0.80) | 0.95 (0.92-0.97) | 6.04 (4.74-8.06) | 0.12 (0.07-0.20) |
| Ravanbakhsh-F4 | 126 | 281 | 43 | 17 | 0.13 (0.10-0.17) | 0.12 (0.08-0.18) | 0.75 (0.67-0.81) | 0.94 (0.91-0.96) | 6.64 (5.10-9.09) | 0.14 (0.08-0.20) |
| Roth | 124 | 273 | 51 | 19 | 0.16 (0.12-0.20) | 0.13 (0.09-0.20) | 0.71 (0.64-0.77) | 0.93 (0.90-0.96) | 5.51 (4.33-7.26) | 0.16 (0.09-0.23) |
| Green and King (G&K) | 105 | 298 | 26 | 38 | 0.08 (0.06-0.12) | 0.27 (0.20-0.34) | 0.8 (0.73-0.86) | 0.89 (0.85-0.92) | 9.15 (6.47-13.94) | 0.29 (0.21-0.37) |
| Kandhro-1 | 134 | 194 | 130 | 9 | 0.4 (0.35-0.46) | 0.06 (0.03-0.12) | 0.51 (0.45-0.57) | 0.96 (0.92-0.98) | 2.34 (2.04-2.70) | 0.11 (0.04-0.18) |
| Sirachainan | 85 | 303 | 21 | 58 | 0.06 (0.04-0.10) | 0.41 (0.33-0.49) | 0.8 (0.72-0.87) | 0.84 (0.80-0.87) | 9.17 (6.15-15.47) | 0.43 (0.34-0.52) |
| Ravanbakhsh-F3 | 107 | 284 | 40 | 36 | 0.12 (0.09-0.16) | 0.25 (0.19-0.33) | 0.73 (0.65-0.79) | 0.89 (0.85-0.92) | 6.06 (4.56-8.44) | 0.29 (0.21-0.38) |
| Bessman | 132 | 195 | 129 | 11 | 0.4 (0.35-0.45) | 0.08 (0.04-0.13) | 0.51 (0.45-0.57) | 0.95 (0.91-0.97) | 2.32 (2.02-2.68) | 0.13 (0.06-0.21) |
| Kandhro-2 | 94 | 292 | 32 | 49 | 0.10 (0.07-0.14) | 0.34 (0.27-0.42) | 0.75 (0.66-0.81) | 0.86 (0.81-0.89) | 6.66 (4.86-9.79) | 0.38 (0.30-0.47) |
| Ricerca | 94 | 292 | 32 | 49 | 0.10 (0.07-0.14) | 0.34 (0.27-0.42) | 0.75 (0.66-0.81) | 0.86 (0.82-0.89) | 6.66 (4.86-9.79) | 0.38 (0.30-0.47) |
| Plengsuree | 94 | 292 | 32 | 49 | 0.10 (0.07-0.14) | 0.34 (0.27-0.42) | 0.75 (0.66-0.81) | 0.86 (0.82-0.89) | 6.66 (4.86-9.79) | 0.38 (0.30-0.47) |
| Matos and Carvalho (M&C) | 108 | 275 | 49 | 35 | 0.15 (0.12-0.19) | 0.24 (0.18-0.32) | 0.69 (0.61-0.76) | 0.89 (0.85-0.92) | 4.99 (3.87-6.84) | 0.29 (0.20-0.38) |
| Zaghloul-1 | 95 | 290 | 34 | 48 | 0.10 (0.08-0.14) | 0.34 (0.27-0.42) | 0.74 (0.65-0.80) | 0.86 (0.82-0.89) | 6.33 (4.71-9.34) | 0.38 (0.29-0.46) |
| Ravanbakhsh-F2 | 93 | 285 | 39 | 50 | 0.12 (0.09-0.16) | 0.35 (0.28-0.43) | 0.7 (0.62-0.78) | 0.85 (0.81-0.88) | 5.4 (4.03-7.65) | 0.4 (0.31-0.49) |
| Sargolzaie | 37 | 312 | 12 | 106 | 0.04 (0.02-0.06) | 0.74 (0.66-0.81) | 0.76 (0.62-0.85) | 0.75 (0.70-0.79) | 6.99 (4.01-15.04) | 0.77 (0.69-0.85) |
| Zaghloul-2 | 80 | 278 | 46 | 63 | 0.14 (0.11-0.18) | 0.44 (0.36-0.52) | 0.63 (0.55-0.71) | 0.82 (0.77-0.85) | 3.94 (2.95-5.53) | 0.51 (0.42-0.61) |
| TI (MCHD) | 92 | 251 | 73 | 51 | 0.23 (0.18-0.27) | 0.36 (0.28-0.44) | 0.56 (0.48-0.63) | 0.83 (0.78-0.87) | 2.86 (2.28-3.65) | 0.46 (0.36-0.57) |
| Pornprasert | 89 | 252 | 72 | 54 | 0.22 (0.18-0.27) | 0.38 (0.30-0.46) | 0.55 (0.48-0.63) | 0.82 (0.78-0.86) | 2.8 (2.22-3.60) | 0.49 (0.38-0.59) |
| Cruise | 36 | 304 | 20 | 107 | 0.06 (0.04-0.09) | 0.75 (0.67-0.81) | 0.64 (0.51-0.76) | 0.74 (0.70-0.78) | 4.08 (2.44-7.33) | 0.8 (0.72-0.88) |
| Huber-Herklotz | 107 | 147 | 177 | 36 | 0.55 (0.49-0.60) | 0.25 (0.19-0.33) | 0.38 (0.32-0.43) | 0.8 (0.74-0.85) | 1.37 (1.19-1.57) | 0.55 (0.39-0.74) |
| SVM | 138 | 304 | 20 | 5 | 0.062 (0.04-0.09) | 0.04 (0.02-0.08) | 0.87 (0.81-0.92) | 0.98 (0.96-0.99) | 15.63 (10.98-26.78) | 0.04 (0.01-0.07) |
| ADA | 139 | 297 | 27 | 4 | 0.08 (0.058-0.12) | 0.03 (0.01-0.07) | 0.84 (0.77-0.89) | 0.99 (0.97-0.99) | 11.67 (8.56-17.75) | 0.03 (0.01-0.06) |
| GB | 139 | 296 | 28 | 4 | 0.09 (0.06-0.12) | 0.03 (0.01-0.07) | 0.83 (0.77-0.88) | 0.99 (0.97-0.99) | 11.25 (8.24-16.86) | 0.03 (0.01-0.06) |
| LR | 138 | 301 | 23 | 5 | 0.07 (0.05-0.10) | 0.04 (0.02-0.08) | 0.86 (0.79-0.90) | 0.98 (0.96-0.99) | 13.59 (9.78-21.51) | 0.04 (0.01-0.07) |
| RF | 134 | 311 | 13 | 9 | 0.04 (0.02-0.07) | 0.06 (0.03-0.12) | 0.91 (0.85-0.95) | 0.97 (0.95-0.99) | 23.35 (15.34-46.29) | 0.07 (0.03-0.11) |
| XGB | 136 | 307 | 17 | 7 | 0.05 (0.03-0.08) | 0.05 (0.02-0.10) | 0.89 (0.83-0.93) | 0.98 (0.95-0.99) | 18.13 (12.35-31.09) | 0.05 (0.02-0.09) |
| MLP | 137 | 303 | 21 | 6 | 0.06 (0.04-0.10) | 0.04 (0.02-0.09) | 0.87 (0.81-0.91) | 0.98 (0.96-0.99) | 14.78 (10.49-24.30) | 0.04 (0.01-0.08) |
| LGBM | 135 | 307 | 17 | 8 | 0.05 (0.03-0.08) | 0.06 (0.03-0.11) | 0.89 (0.83-0.90) | 0.97 (0.95-0.99) | 17.99 (12.37-32.32) | 0.06 (0.02-1.00) |
| LDA | 133 | 309 | 15 | 10 | 0.05 (0.03-0.08) | 0.07 (0.04-0.12) | 0.90 (0.84-0.94) | 0.97 (0.94-0.98) | 20.09 (13.44-38.23) | 0.07 (0.03-0.12) |
| NB | 137 | 296 | 28 | 6 | 0.09 (0.06-0.12) | 0.04 (0.02-0.09) | 0.83 (0.77-0.88) | 0.98 (0.96-0.99) | 11.09 (8.21-17.17) | 0.05 (0.01-0.08) |
| KNN | 137 | 295 | 29 | 6 | 0.09 (0.06-0.13) | 0.04 (0.02-0.09) | 0.83 (0.76-0.88) | 0.98 (0.96-0.99) | 10.7 (8.00-16.08) | 0.05 (0.01-0.09) |
| DT | 123 | 305 | 19 | 20 | 0.06 (0.04-0.09) | 0.14 (0.09-0.21) | 0.87 (0.80-0.91) | 0.94 (0.91-0.96) | 14.67 (10.19-25.32) | 0.15 (0.09-0.21) |
